# Behavioral and psychosocial factors associated with sugar-sweetened beverage consumption among Korean adolescents: a path analysis using the 2022 Korea Youth Risk Behavior Survey

**DOI:** 10.4178/epih.e2025047

**Published:** 2025-08-21

**Authors:** Hye-Young Park, Soo Rack Ryu, Hoon-Ki Park, Hwan-Sik Hwang, Kye-Yeung Park

**Affiliations:** 1 Department of Family Medicine, Hanyang University College of Medicine, Seoul, Korea; 2 Biostatistical Consulting and Research Lab, Medical Research Collaborating Center, Hanyang University, Seoul, Korea

**Keywords:** Sugar-sweetened beverages, Adolescent, Eating, Obesity, Screen time

## Abstract

**OBJECTIVES:**

Rising obesity rates among adolescents are a major global health concern and are closely linked to the consumption of sugar-sweetened beverages (SSBs). This study aimed to identify key behavioral and psychosocial factors influencing SSB consumption among adolescents.

**METHODS:**

This cross-sectional study analyzed data from the 2022 Korea Youth Risk Behavior Survey, which included 49,548 participants aged 12-18 years. Information on SSB consumption frequency, socio-demographic characteristics, eating habits, sedentary behaviors, and other health-related factors was collected through self-administered questionnaires. Path analysis was used to model SSB consumption and estimate the direct and indirect effects of modifiable factors.

**RESULTS:**

Male students, current alcohol drinkers, those with higher frequencies of fast-food or late-night snack consumption, and heavy smartphone users were more likely to frequently consume SSBs. Fast-food intake had the strongest direct effect on SSB consumption (B=0.3884), while nighttime eating showed a substantial direct effect (B=0.1437) and mediated 21.7% of the relationship between fast-food intake and SSB consumption. Leisure sitting time exerted both direct (B=0.0741) and indirect effects on SSB intake, mediated through watching mukbang, smartphone use, fast-food consumption, and nighttime eating. Self-perceived health status was negatively associated with SSB consumption (B=-0.0619), with indirect effects mediated by fast-food intake and nighttime eating.

**CONCLUSIONS:**

Among Korean adolescents, SSB consumption was strongly associated with unhealthy eating patterns and prolonged leisure sitting time. Increased fast-food consumption, nighttime eating, watching mukbang, smartphone use, and negative self-perceived health status not only directly influenced SSB intake, but also acted as mediating factors.

## GRAPHICAL ABSTRACT

[Fig f2-epih-47-e2025047]

## Key Message

Unhealthy eating behaviors and excessive screen time are strongly linked to sugar-sweetened beverage (SSB) consumption among Korean adolescents. Fast-food intake and nighttime snacking act as major mediators, suggesting that reducing these behaviors could significantly decrease SSB intake. These findings provide evidence to guide targeted public health interventions for adolescents.

## INTRODUCTION

Excessive consumption of sugar-sweetened beverages (SSBs) among adolescents has become a critical public health concern due to its association with obesity and chronic diseases such as type 2 diabetes and cardiovascular disorders [1-4]. The World Health Organization (WHO) has reported that SSB overconsumption contributes to excessive caloric intake and increases the risk of obesity. A review covering 51 countries found that SSB consumption among adolescents frequently exceeded WHO recommendations [5]. In Korea, several studies have highlighted the high prevalence of SSB intake and its associated health risks [6-8]. The 2022 Korea Youth Risk Behavior Survey (KYRBS) showed that more than 88% of adolescents consumed SSBs at least once in the previous week, and nearly 15% reported daily consumption, underscoring the magnitude of the issue.

Socio-demographic characteristics and screen-related behaviors are significant determinants of adolescent SSB consumption. Park et al. [9] found notable associations with male sex, frequent fast-food consumption, and increased screen time. Cha et al. [10] reported that adolescents engaging in ≥6 hours of media use per day had higher odds of nighttime eating, a behavior linked to unhealthy dietary patterns and adverse health outcomes [11-15].

Recently, the growing popularity of food-related digital content, particularly “mukbang” and “cookbang” (online eating and cooking shows), has raised concerns about its influence on adolescents’ dietary habits. Regular viewers of such content are more likely to consume fast food, engage in late-night snacking, and drink SSBs [16-18]. These tendencies may have been amplified during the coronavirus disease 2019 (COVID-19) pandemic, which increased screen time and disrupted established eating patterns [19]. Given these trends, there is a pressing need to examine modifiable behavioral factors and develop timely preventive interventions.

In addition to behavioral influences, psychosocial factors such as self-perceived health status also play a role in shaping adolescents’ dietary choices [20]. Adolescents with negative perceptions of their health are more likely to follow unhealthier eating patterns, including higher consumption of fast food and sugary beverages. Calabro et al. [21] emphasized that socio-cognitive determinants—such as attitudes and perceived norms—are associated with SSB-related behaviors. Similarly, Mahmoodianfard & Haghighat [22] highlighted the importance of self-efficacy and perceived barriers in shaping youth diets. These findings support the integration of psychosocial and behavioral variables into conceptual models aimed at understanding the pathways leading to SSB consumption.

Therefore, this study aimed to investigate multiple factors influencing SSB consumption among Korean adolescents by addressing both behavioral (e.g., fast-food consumption, nighttime snacking, screen time) and psychosocial (e.g., self-perceived health status) variables. While previous research has examined individual correlates of SSB intake, few studies have explored the complex interrelationships among these factors. Using 2022 KYRBS data, we conducted a path analysis to evaluate both direct and indirect associations, with the goal of informing targeted public health strategies to address SSB-related health risks in adolescents.

## MATERIALS AND METHODS

### Study design and population

This cross-sectional study used data from the 2022 KYRBS, an anonymous, self-reported online survey conducted annually by the Korea Disease Control and Prevention Agency (KDCA) and the Ministry of Education to inform youth health policies. The KYRBS applies a multistage cluster sampling technique involving stratification and clustering. To address the complexity of this survey design, all analyses incorporated sampling weights, stratification, and clustering, as recommended by the KDCA. The survey provides a nationally representative sample of Korean students from 800 schools, covering both public and private institutions across 17 provinces.

The survey was administered online using school computers and mobile devices (tablet PCs and smartphones) in classrooms under teacher supervision. The questionnaire consisted of 114 items across 16 categories, including dietary habits, lifestyle factors, and health-related behaviors. In 2022, the SSB-related question was revised to include sugar-containing beverages, and new questions were added on late-night snacking and viewing eating shows such as mukbang.

Of 56,213 eligible students, 51,850 completed the survey between August 2022 and October 2022. After excluding responses with missing data, 49,548 participants aged 12-18 years were included in the final analysis.

### Rationale for variable selection

The selection of variables was informed by previous empirical research and theoretical models on adolescent health behavior, including the socio-ecological framework and prior studies identifying behavioral and psychosocial predictors of SSB consumption (e.g., fast-food intake, screen time, self-perceived health) [1-4,20]. Variables such as mukbang/cookbang viewing and smartphone use reflect emerging digital lifestyle trends in Korea that influence eating behaviors and sedentary time, which are factors that have become increasingly relevant in the post-pandemic period [16,17,19].

### Sugar-sweetened beverage consumption

The primary outcome was the frequency of SSB intake. Consumption was assessed through the question: “During the last 7 days, how often did you drink SSBs?” Examples provided included carbonated drinks (excluding zero-sugar varieties), energy drinks (e.g., Hot Six, Red Bull, Bacchus), isotonic beverages (e.g., Gatorade, Pocari Sweat), fruit juice (e.g., Sunkist drink, Capri-Sun), coffee drinks (e.g., coffee mix, canned coffee), and sweetened milk (e.g., chocolate milk, banana milk). Respondents chose from 7 options: (1) not at all, (2) 1-2 times/wk, (3) 3-4 times/wk, (4) 5-6 times/wk, (5) once daily, (6) twice daily, and (7) 3 or more times daily.

### Other variables

#### Fast food consumption and nighttime eating

Participants reported the number of times they consumed fast food and nighttime snacks during the past week. Fast food included items such as pizza, burgers, and fried chicken. Nighttime eating was defined in the KYRBS as meals or snacks consumed after dinner, excluding small items like fruit or milk, which were not considered markers of unhealthy eating. In the path analysis, both variables were treated as continuous measures (times/wk), while for descriptive statistics they were categorized (e.g., ≥3 times/wk). The ≥3 times/wk threshold followed KYRBS criteria, which are regularly used by the KDCA to monitor adolescent dietary behaviors.

#### Leisure sitting time

Average daily sitting time was measured by the question: “In the last 7 days, how many hours on average per day have you spent sitting?” Responses were categorized into (1) study-related sitting (e.g., school, homework, educational broadcasts) and (2) leisure sitting (e.g., watching TV, gaming, internet browsing, chatting, commuting). Weighted averages for weekdays (5/7) and weekends (2/7) were calculated. Based on prior research indicating a stronger association between screen-based sedentary behavior and SSB intake [12,13], the path analysis focused on leisure sitting time, which was treated as a continuous variable.

#### Screen time

Screen-related variables included: (1) watching mukbang or cookbang and (2) smartphone usage time. The 2022 KYRBS introduced questions on mukbang and cookbang for the first time. Participants were asked how often they watched eating broadcasts such as mukbang, which are videos in which individuals eat food while interacting with viewers through virtual comments on platforms like YouTube or other internet broadcasts [23], and cookbang, which are online cooking shows. Popular platforms included Instagram, Facebook, TikTok, YouTube, AfreecaTV, KakaoTV, and Twitch. Daily smartphone use was assessed with the question: “In the last 7 days, how many hours on average per day have you used a smartphone?” Weighted averages for weekdays and weekends (5/7 and 2/7, respectively) were calculated.

Smartphone use was categorized as <4 hr/day or ≥4 hr/day, based on evidence that adverse health outcomes become more common when daily screen time exceeds 3-4 hours, and this variable was analyzed using the chi-square test [24-28]. Large-scale studies have also found that many adolescents use smartphones for more than 4 hr/day [29]. Although earlier recommendations often suggested limiting screen time to 2 hr/day, this benchmark may no longer reflect current usage patterns. Thus, the 4-hour threshold was selected to align with recent evidence and prevailing media consumption trends. Continuous values were used in correlation and path analyses to preserve variability.

#### Self-perceived health status

Participants assessed their general health by responding to the question: “How do you perceive your health status in general?” Responses ranged from 1 (very healthy) to 5 (very unhealthy).

### Statistical analysis

The basic characteristics of the study population were examined by calculating the absolute and relative frequencies of socioeconomic, dietary, and lifestyle variables according to levels of SSB consumption. All analyses accounted for the complex survey design of the KYRBS, incorporating sampling weights, stratification, and clustering through the complex samples procedures in SPSS version 26.0 (IBM Corp., Armonk, NY, USA).

All categorical variables in Table 1—including fast-food and vegetable consumption—were dichotomized using established thresholds adopted from the KYRBS for monitoring adolescent health behaviors. As noted earlier, these cutoff points have been widely applied in previous research utilizing KYRBS data and serve as practical benchmarks aligned with public health recommendations. Although the WHO does not specify explicit weekly frequency limits, the thresholds used in this study were consistent with WHO dietary guidelines for adolescents, ensuring comparability with prior epidemiological studies [7,16,30].

Several demographic and health-related factors, such as smoking status, alcohol consumption, and household economic status, were retained in Table 1 to provide a comprehensive description of the study population, although they were not included in the path analysis.

To investigate complex interrelationships among variables, structural equation modeling with path analysis was conducted using LISREL version 8.52 (Scientific Software International Inc., Lincolnwood, IL, USA). While structural equation modeling was performed in LISREL and mediation analysis was conducted via the PROCESS macro, these methods do not fully incorporate complex sampling adjustments; therefore, results should be interpreted with appropriate caution. Correlation analysis was used to assess the strength and direction of associations between variables, and a hypothesized path diagram was developed to depict both direct and indirect pathways influencing SSB consumption [31]. In the structural model, household economic status and self-rated health were reverse-coded to ensure consistent interpretation across variables.

Model fit was evaluated using standard indices, including the root mean square error of approximation (RMSEA), goodness-of-fit index (GFI), comparative fit index (CFI), and normed fit index (NFI). Mediation effects were tested using the PROCESS macro for SPSS (version 3.5; Hayes, 2013), applying model 6 (a serial dual mediation model). The significance of indirect effects was assessed through bootstrap resampling with 5,000 samples and 95% confidence intervals (CIs). This multi-method analytic strategy provided a comprehensive evaluation of both the direct and indirect mechanisms linking lifestyle and psychosocial factors to SSB consumption.

### Ethics statement

This study was approved by the Institutional Review Board of Hanyang University Hospital, Seoul, Korea (No. HYUH 2024-08-018), and adhered to the ethical guidelines of the World Medical Association’s Declaration of Helsinki. The board granted an exemption from informed consent because all data used in the analysis were anonymous and de-identified.

## RESULTS

### Baseline characteristics

Participants were categorized into 3 groups based on weekly SSB intake. Of the 49,548 participants, 3,233 (6.5%) reported no SSB consumption in the past 7 days, 39,032 (78.8%) consumed SSBs 1-6 times/wk (moderate frequency), and 7,283 (14.7%) consumed them more than once daily (high frequency).

Males consumed SSBs more frequently than females (p<0.001), particularly in the high-frequency group (59.0% males vs. 41.0% females). Significant differences in SSB intake were also observed by academic performance, household income, smoking status, and alcohol use between the moderate-consumption and high-consumption groups (p<0.001).

High-frequency consumers were more likely to watch mukbang (69.9%), use smartphones for more than 4 hr/day (64.3%), and engage in late-night snacking (71.1%) (p<0.001). Frequent fast-food consumption, breakfast skipping, and prolonged leisure sitting time were also associated with higher SSB intake (p<0.001), underscoring the link between SSB consumption and other unhealthy behaviors in adolescents.

Interestingly, the mean body mass index (BMI) was highest among adolescents who had not consumed SSBs in the past 7 days (21.48±0.08 kg/m^2^) and lowest among those in the high-frequency group (21.06±0.06 kg/m^2^), with statistically significant differences across groups (p<0.001). The prevalence of BMI ≥ 25 kg/m^2^ was significantly different across SSB consumption groups (p<0.001). Contrary to expectations, the proportion was highest among non-SSB consumers (17.7%) and lowest among daily consumers (14.6%).

### Correlation between sugar-sweetened beverage consumption and other lifestyle factors

As shown in Table 2, SSB intake was positively correlated with watching mukbang (r=0.085), smartphone use (r=0.122), leisure sitting time (r=0.074), fast-food consumption (r=0.301), and nighttime eating (r=0.257). Self-perceived health was negatively correlated with SSB intake (r=-0.058). All correlations were statistically significant (p<0.001). Fast-food intake (r=0.301) and nighttime eating (r=0.257) showed the strongest associations.

### Path analysis of lifestyle factors influencing sugar-sweetened beverage consumption

**Figure 1.**
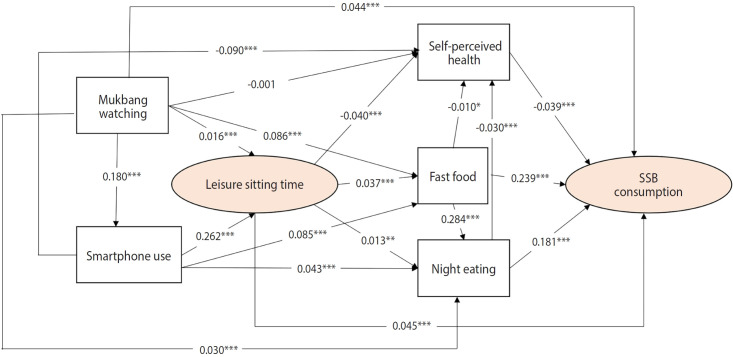
Results of path analysis. Path analysis model of leisure sitting time, watching mukbang, smartphone use, self-perceived health, fast food consumption, nighttime eating, and sugar-sweetened beverage (SSB) consumption. *p<0.05, **p<0.01, ***p<0.001.

Figure 1 shows that fast-food consumption exerted the strongest direct effect on SSB intake (r=0.239), followed by nighttime snacking (r=0.181). Fast-food consumption was also indirectly associated with SSB intake via increased nighttime snacking frequency (r=0.284). Smartphone use was significantly associated with leisure sitting time (r=0.262), which in turn was linked to higher fast-food intake and nighttime eating. These behaviors were related to poorer self-perceived health, which was further associated with higher SSB consumption.

Subgroup path analyses stratified by sex (Supplementary Materials 1 and 2) and school level (Supplementary Materials 3 and 4) demonstrated that the effects of fast-food consumption and nighttime eating on SSB intake were consistently significant across all groups, indicating robust associations regardless of sex or school level. However, some subgroup-specific patterns emerged:

(1) The association between self-perceived poor health and SSB consumption was more pronounced among females and high school students.
(2) The impact of leisure sitting time on SSB intake was stronger among males and middle school students.
(3) A significant link between mukbang viewing and nighttime snacking was found only in females and middle school students, but not among high school students.
(4) Mukbang viewing was significantly associated with leisure sitting time among male students (β=0.045, p<0.05), while no such association was observed in females.

Supplementary Material 5 summarizes key path coefficients by sex and school level, highlighting both consistent and group-specific behavioral pathways related to SSB intake.

Model fit indices indicated an overall acceptable fit (RMSEA=0.064; GFI=0.999; CFI=0.991; NFI=0.991). GFI, CFI, and NFI suggested an excellent fit, while RMSEA was within the acceptable range (Table 3) [29].

### Direct and indirect effects on sugar-sweetened beverage consumption based on path analysis

Table 4 presents the regression coefficients (B) for each pathway. To assess mediation strength, indirect effect ratios were calculated as: B of indirect pathways/B of direct pathways ×100.

Leisure sitting time increased SSB intake both directly (B=0.0741; 95% CI, 0.0452 to 0.1031) and indirectly via smartphone use (B=0.0291) and fast-food intake (B=0.0124). Smartphone use accounted for 39.28% of the indirect effect, while fast-food intake accounted for 16.75%.

Fast-food consumption had the strongest direct effect (B=0.3884; 95% CI, 0.3748 to 0.4020), indicating that each unit increase in fast-food intake corresponded to a 0.3884-unit rise in SSB intake. Nighttime eating was the most influential mediator of this relationship (B=0.0844; 21.73% indirect effect). Fast-food intake also mediated the relationship between nighttime eating and SSB intake (39.08% indirect effect). Nighttime eating itself had a notable direct effect (B=0.1437; 95% CI, 0.1369 to 0.1505).

Self-perceived health status demonstrated both negative direct and indirect effects. Poorer health perception increased fast-food intake, which in turn raised SSB consumption (B=-0.0111), and also had a direct negative association with SSB intake (B=-0.0619; 95% CI, -0.0739 to -0.0500).

## DISCUSSION

Our findings highlight the multifaceted associations between adolescents’ dietary behaviors, screen-based leisure activities, and health perceptions, all of which demonstrated significant relationships with SSB consumption. Using path analysis, we identified both direct and indirect associations among these variables, with fast-food intake and nighttime eating emerging as key mediating factors.

Fast-food consumption was the strongest predictor of SSB intake, potentially due to its high sodium and fat content, which may stimulate cravings for sweetened beverages to offset the richness of the meal [7]. Additionally, marketing strategies, such as bundling sugary drinks with fast-food items, likely reinforce this consumption pattern among adolescents [7]. Beyond its direct effect, fast-food intake also served as a mediator, linking sedentary behaviors and nighttime snacking to increased SSB consumption. Nighttime eating was directly associated with SSB intake and mediated the relationship between fast-food consumption and SSBs, reinforcing prior evidence that connects nighttime eating with increased fast-food and SSB intake, as well as higher BMI in adolescents [32-34].

Screen-based behaviors—particularly prolonged smartphone use and mukbang viewing—were positively associated with extended leisure sitting time. In our analysis, these variables were examined separately, revealing that while both were linked to greater fast-food consumption and nighttime snacking, mukbang viewing showed a stronger direct association with SSB intake. Mukbang viewing was also positively correlated with smartphone use, suggesting that food-related digital content may act as an environmental trigger that amplifies both media engagement and unhealthy dietary behaviors. Given that mukbang is often consumed during evening hours, it may stimulate food cravings and encourage late-night snacking and SSB intake [33]. This aligns with findings by Hernandez et al. [32], who reported that 21% of Korean adolescents engage in nighttime snacking, a behavior linked to higher BMI Z-scores. Similar associations between screen time and SSB consumption have been reported in Brazil [31] and Spain [35].

Self-perceived poor health was associated with higher fast-food and nighttime snack consumption, which in turn was linked to greater SSB intake. These results are consistent with findings from Osera et al. [36] and a large-scale 2022 meta-analysis [21]. Notably, adolescents with negative health perceptions were also more likely to engage in prolonged screen-based sedentary behaviors, highlighting the role of psychosocial factors in reinforcing unhealthy dietary patterns through increased sedentary media use. Unlike earlier studies that primarily examined isolated associations between SSB consumption and individual factors such as fast-food intake or screen time [9,11,18,20,35], our study addresses this gap by illustrating how behavioral and psychosocial factors interact to influence dietary habits that drive higher SSB intake.

Global public health authorities have underscored the need to address excessive SSB consumption in adolescents. The WHO has identified high SSB intake as a major contributor to increased free sugar consumption, which is directly associated with obesity risk [5,30]. In a comprehensive review, Calcaterra et al. [37] concluded that the primary physiological mechanism linking SSB consumption to weight gain is the beverage’s minimal effect on satiety, which results in incomplete compensatory reductions in caloric intake at subsequent meals. This process contributes to a net caloric surplus and fat accumulation, particularly among youth. These findings suggest that modifiable lifestyle behaviors—including frequent fast-food consumption, late-night snacking, and prolonged screen exposure—should be prioritized in youth obesity prevention strategies.

Unexpectedly, our analysis revealed that the prevalence of adolescents with BMI ≥25 kg/m^2^ was highest among non-SSB consumers and lowest among daily SSB consumers. While statistically significant (p<0.001), this result should be interpreted with caution. Possible explanations include reverse causality, whereby overweight adolescents may have intentionally reduced SSB intake, as well as unmeasured confounders such as differences in physical activity or metabolic factors [38]. Longitudinal studies are needed to clarify this relationship.

This study has several strengths, including a robust analytical framework and the use of a nationally representative adolescent sample. However, limitations should be noted. First, the cross-sectional design prevents causal inference, and the associations reported should be interpreted as correlational. Second, self-reported measures may be subject to recall bias and social desirability bias. Third, physical activity was not assessed, which could confound the observed associations between sedentary behavior and SSB intake. Fourth, SSB consumption was measured using a single composite item that combined 6 beverage types, preventing beverage-specific analyses. Fifth, newly introduced variables such as mukbang and cookbang viewing have not yet been fully validated in the KYRBS, and their psychometric properties require further evaluation. Sixth, the KYRBS did not provide specific examples of nighttime snacks, and its exclusion of certain items such as fruit or milk was not fully justified, potentially affecting consistency in responses. Finally, generalizability beyond the Korean context may be limited.

Overall, this study presents a behavioral framework in which fast-food consumption acts as both a direct and indirect driver of SSB intake, underscoring the importance of public health campaigns that limit fast-food accessibility and promote healthier alternatives. The observed subgroup differences further suggest that interventions should be tailored to sex-level and school-level-specific behavioral patterns. For example, reducing screen time, limiting mukbang viewing, and promoting healthier eating behaviors may require different strategies for male and female students, as well as for middle school versus high school populations. Such targeted approaches could enhance the effectiveness of public health interventions aimed at improving adolescent dietary behaviors.

## Figures and Tables

**Figure f2-epih-47-e2025047:**
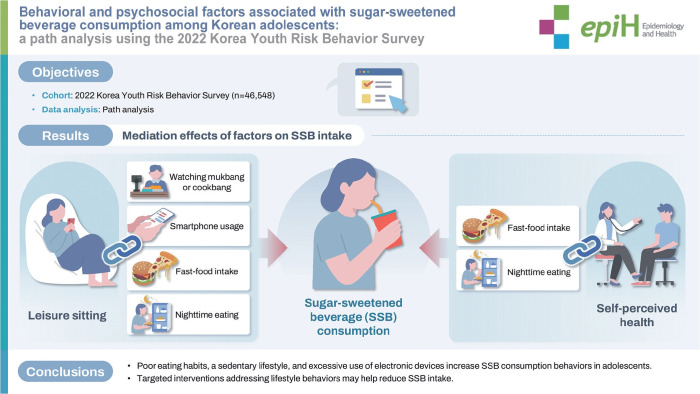


**Table 1.** General characteristics of the study population according to SSB consumption frequency

Characteristics	Total (n=49,548)	None in last 7 days (n=3,233)	1–6 times/wk (n=39,032)	≥1 time/day (n=7,283)	p-value
Age (mo)	187.94±0.31	186.98±0.52	187.65±0.32	189.91±0.41	<0.001
Sex					<0.001
Male	51.7	48.0	50.6	59.0	
Female	48.3	52.0	49.4	41.0	
Grade					<0.001
Middle school	51.8	54.8	52.3	47.5	
High school	48.2	45.2	47.7	52.5	
BMI (kg/m^2^)					<0.001
<25	83.6	82.3	83.4	85.4	
≥25	16.4	17.7	16.6	14.6	
Mean	21.35±0.04	21.48±0.08	21.40±0.04	21.06±0.06	
Household economic status					<0.001
High	43.4	43.5	43.3	44.1	
Middle	46.2	45.2	46.6	44.4	
Low	10.4	11.3	10.1	11.5	
Current smoking					<0.001
No	91.3	94.1	91.8	87.6	
Yes	8.7	5.9	8.2	12.4	
Current alcohol consumption					<0.001
No	66.1	74.4	66.8	58.7	
Yes	33.9	25.6	33.2	41.3	
Fast food consumption (times/wk)^1^					<0.001
<3	73.0	89.7	74.6	56.9	
≥3	27.0	10.3	25.4	43.1	
Vegetable consumption					<0.001
<3	22.9	22.4	22.5	25.3	
≥3	77.1	77.6	77.5	74.7	
Nighttime eating (day/wk)^1^					<0.001
<1	40.0	65.1	40.0	28.9	
≥1	60.0	34.9	60.0	71.1	
Watching mukbang or cookbang^1^					<0.001
No	29.5	39.6	28.5	30.1	
Yes	70.5	60.4	71.5	69.9	
Smartphone use (hr/day)^1^					<0.001
<4	42.3	49.3	42.9	35.7	
≥4	57.7	50.7	57.1	64.3	
Sitting time for studying (hr/day)					0.153
<6.5	49.7	49.2	49.5	50.8	
≥6.5	50.3	50.8	50.5	49.2	
Sitting time for leisure^1^					<0.001
<3.6	57.3	61.1	57.8	52.9	
≥3.6	42.7	38.9	42.2	47.1	

Values are presented as weighted percentages for categorical variables and weighted mean±SE for continuous variables. SSB, sugar-sweetened beverage; SE, standard error; BMI, body mass index.

1 Variables retained for path analysis.

**Table 2.** Correlations between 6 modifiable variables and sugar-sweetened beverage (SSB) consumption

		1	2	3	4	5	6	7
1. Watching mukbang or cookbang	r	1.000						
	p-value							
2. Smartphone use	r	0.180	1.000					
	p-value	<0.001						
3. Leisure sitting time	r	0.064	0.265	1.000				
	p-value	<0.001	<0.001					
4. Self-perceived health	r	-0.022	-0.104	-0.065	1.000			
	p-value	<0.001	<0.001	<0.001				
5. Fast food intake	r	0.103	0.110	0.065	-0.031	1.000		
	p-value	<0.001	<0.001	<0.001	<0.001			
6. Nighttime eating	r	0.067	0.083	0.044	-0.042	0.292	1.000	
	p-value	<0.001	<0.001	<0.001	<0.001	<0.001		
7. SSB intake	r	0.085	0.122	0.074	-0.058	0.301	0.257	1.000
	p-value	<0.001	<0.001	<0.001	<0.001	<0.001	<0.001	

**Table 3.** Goodness-of-fit statistics for the final model

	Model result	Cutoff value	Annotation
*χ*² (p-value)	202.907 (p<0.001)	p>0.05	Not fit
df	1		
*χ*²/df	202.907	≤3.00	Not fit
		≤0.05: Good	
RMR	0.011	0.05–≤0.08: Not bad	Fit
		≤0.05: Good	
RMSEA	0.064	≤0.08: Not bad	Fit
		≤0.10: Moderate	
GFI	0.999	≥0.90	Fit
AGFI	0.967	≥0.90	Fit
NNFI	0.804	≥0.90	Not fit
NFI	0.991	≥0.90	Fit
CFI	0.991	≥0.90	Fit
IFI	0.991	≥0.90	Fit
RFI	0.803	≥0.90	Not fit

df, degrees of freedom; RMR, root mean square residual; RMSEA, root mean square error of approximation; GFI, goodness-of-fit index; AGFI, adjusted goodness-of-fit index; NNFI, non-normed fit index; NFI, normed fit index; CFI, comparative fit index; IFI, incremental fit index; RFI, relative fit index.

**Table 4.** Direct and indirect effects from path analysis

Effects	Relationship	B	Boot SE	95% CI	
				LL	UL
Direct	Leisure sitting time → SSB intake	0.0741	0.0079	0.0452	0.1031
Indirect	Leisure sitting time → Self-perceived health → SSB intake	0.0026	0.0003	0.0019	0.0033
	Leisure sitting time → Nighttime eating → SSB intake	0.0076	0.0009	0.0059	0.0094
	Leisure sitting time → Fast food intake → SSB intake	0.0124	0.0012	0.0101	0.0147
	Leisure sitting time → Self-perceived health → Nighttime eating → SSB intake	0.0005	0.0001	0.0003	0.0006
	Leisure sitting time → Self-perceived health → Fast food intake → SSB intake	0.0003	0.0001	0.0001	0.0004
	Leisure sitting time → Nighttime eating → Fast food intake → SSB intake	0.0029	0.0003	0.0023	0.0036
	Leisure sitting time → Self-perceived health → Nighttime eating → Fast food intake → SSB intake	0.0002	0.0000	0.0001	0.0002
	Leisure sitting time → Smartphone use → SSB intake	0.0291	0.0015	0.0263	0.0320
	Leisure sitting time → Watching mukbang or cookbang → SSB intake	0.0051	0.0005	0.0042	0.0061
	Leisure sitting time → Fast food intake → Self-perceived health → SSB intake	0.0001	0.0000	0.0000	0.0001
	Leisure sitting time → Nighttime eating →Self-perceived health → SSB intake	0.0001	0.0000	0.0000	0.0001
Direct	Fast food intake → SSB intake	0.3884	0.0070	0.3748	0.4020
Indirect	Fast food intake → Self-perceived health → SSB intake	0.0021	0.0004	0.0014	0.0030
	Fast food intake → Nighttime eating → SSB intake	0.0844	0.0028	0.0791	0.0899
	Fast food intake → Self-perceived health → Nighttime eating → SSB intake	0.0003	0.0001	0.0002	0.0004
Direct	Nighttime eating → SSB intake	0.1437	0.0034	0.1369	0.1505
Indirect	Nighttime eating → Self-perceived health → SSB intake	0.0014	0.0002	0.0010	0.0019
	Nighttime eating → Fast food intake → SSB intake	0.0561	0.0016	0.0531	0.0591
	Nighttime eating → Self-perceived health → Fast food intake → SSB intake	0.0002	0.0000	0.0001	0.0002
Direct	Self-perceived health → SSB intake	-0.0619	0.0061	-0.0739	-0.0500
Indirect	Self-perceived health → Fast food intake → SSB intake	-0.0111	0.0018	-0.0147	-0.0077
	Self-perceived health → Nighttime eating → SSB intake	-0.0087	0.0012	-0.0113	-0.0064
	Self-perceived health → Fast food intake → Nighttime eating → SSB intake	-0.0024	0.0004	-0.0032	-0.0017

SE, standard error; CI, confidence interval; LL, lower limit; UL, upper limit; SSB, sugar-sweetened beverage.

## Supplementary materials

Supplementary Material 1. Results of path analysis (male). Path analysis model of leisure sitting time, watching Mukbang, smartphone use, self-perceived health, fast food consumption, nighttime eating, and SSB consumption.

Supplementary Material 2. Results of path analysis (female). Path analysis model of leisure sitting time, watching Mukbang, smartphone use, self-perceived health, fast food consumption, nighttime eating, and SSB consumption.

Supplementary Material 3. Results of path analysis (middle school). Path analysis model of leisure sitting time, watching Mukbang, smartphone use, self-perceived health, fast food consumption, nighttime eating, and SSB consumption.

Supplementary Material 4. Results of path analysis (high school). Path analysis model of leisure sitting time, watching Mukbang, smartphone use, self-perceived health, fast food consumption, nighttime eating, and SSB consumption.

Supplementary Material 5. Key Path coefficients by sex and school level
