# Supplementary material for: Behavioral and psychosocial factors associated with sugar-sweetened beverage consumption among Korean adolescents: a path analysis using the 2022 Korea Youth Risk Behavior Survey
Source: Epidemiol Health. 2025 Aug 21;47:e2025047. doi: 10.4178/epih.e2025047 (PMC12869117; doi:10.4178/epih.e2025047)
Supplement: Supplementary Material 2. — Results of path analysis (female). Path analysis model of leisure sitting time, watching Mukbang, smartphone use, self-perceived health, fast food consumption, nighttime eating, and SSB consumption. [file epih-47-e2025047-Supplementary-2.docx]

**
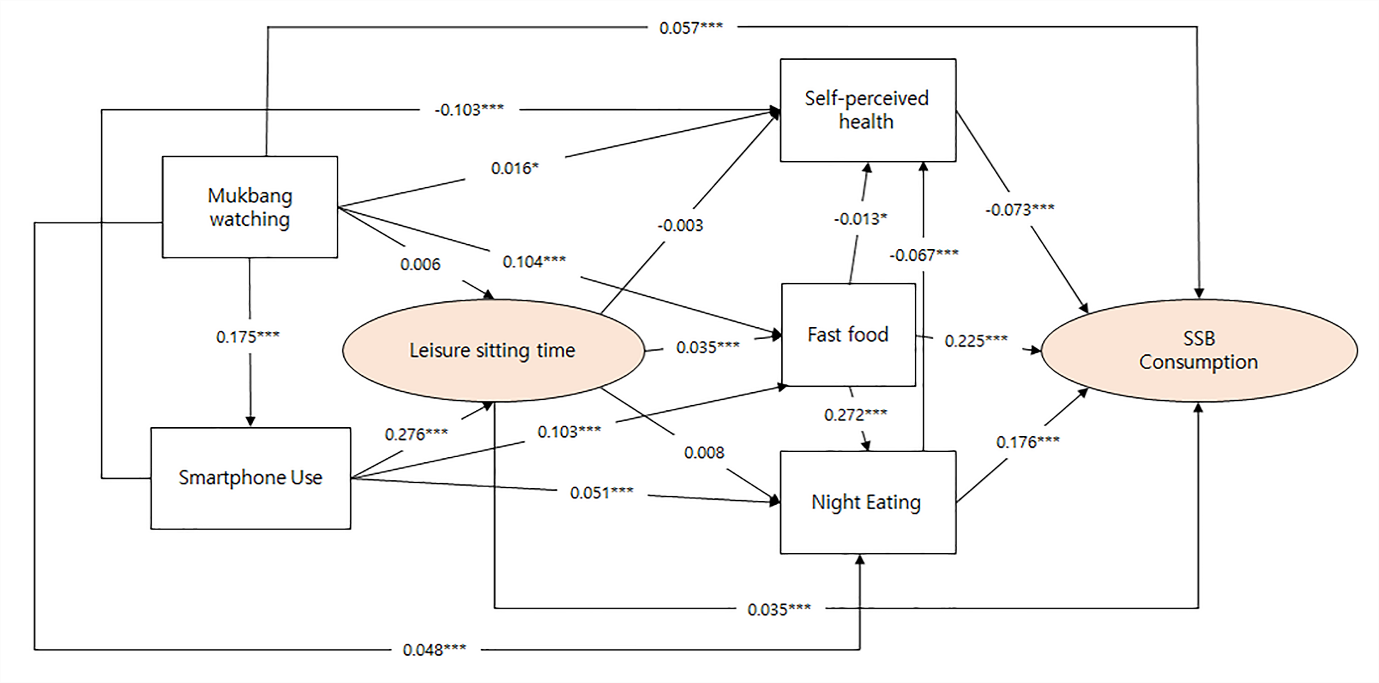
**

**Supplementary Material 2.** Results of path analysis (female). Path analysis model of leisure sitting time, watching Mukbang, smartphone use, self-perceived health, fast food consumption, nighttime eating, and SSB consumption.

^*^p<.05, ^**^p<.01, ^***^p<.001

Abbreviations: SSB, sugar-sweetened beverage
