# Supplementary material for: Behavioral and psychosocial factors associated with sugar-sweetened beverage consumption among Korean adolescents: a path analysis using the 2022 Korea Youth Risk Behavior Survey
Source: Epidemiol Health. 2025 Aug 21;47:e2025047. doi: 10.4178/epih.e2025047 (PMC12869117; doi:10.4178/epih.e2025047)
Supplement: Supplementary Material 5. — Key Path coefficients by sex and school level [file epih-47-e2025047-Supplementary-5.docx]

Supplementary Material 5. Key Path coefficients by sex and school level

| Path | Male | Female | Middle School | High School |
| --- | --- | --- | --- | --- |
| Fast Food → SSB | 0.244^***^ | 0.225^***^ | 0.243^***^ | 0.235^***^ |
| Night Eating → SSB | 0.178^***^ | 0.176^***^ | 0.173^***^ | 0.182^***^ |
| Self-perceived health → SSB | - 0.029^***^ | - 0.073^***^ | - 0.026^***^ | - 0.051^***^ |
| Leisure Sitting Time → SSB | 0.049^***^ | 0.035^***^ | 0.048^***^ | 0.044^***^ |
| Mukbang → SSB | 0.057^***^ | 0.057^***^ | 0.063^***^ | 0.023^***^ |
| Mukbang → Night Eating | 0.024^***^ | 0.048^***^ | 0.051^***^ | 0.009(ns) |
| Mukbang → Fast Food | 0.087^***^ | 0.104^***^ | 0.099^***^ | 0.070^***^ |
| Mukbang → Leisure Sitting Time | 0.045^***^ | 0.006(ns) | 0.013^*^ | 0.020^**^ |
| Mukbang → Smartphone Use | 0.148^***^ | 0.175^***^ | 0.189^***^ | 0.171^***^ |
| Smartphone Use → Fast Food | 0.089^***^ | 0.103^***^ | 0.085^***^ | 0.079^***^ |
| Smartphone Use → Night Eating | 0.048^***^ | 0.051^***^ | 0.054^***^ | 0.017^*^ |
| Smartphone Use → Leisure Sitting Time | 0.265^***^ | 0.276^***^ | 0.268^***^ | 0.263^***^ |
| Fast food → Night eating | 0.287^***^ | 0.272^***^ | 0.270^***^ | 0.294^***^ |
| Fast food → Self-perceived health | - 0.024^***^ | - 0.013^*^ | - 0.010^*^ | - 0.010^*^ |
| Leisure Sitting Time → Fast Food | 0.026^***^ | 0.035^***^ | 0.047^***^ | 0.028^***^ |
| Leisure Sitting Time → Night Eating | 0.010^*^ | 0.008(ns) | 0.019^**^ | 0.016^*^ |
| Leisure Sitting Time → Self-perceived health | - 0.092^***^ | - 0.003(ns) | - 0.047^***^ | - 0.038^***^ |
| Abbreviations: ns = not significant; ^*^p<.05, ^**^p<.01, ^***^p<.001 | | | | |
